# Supplementary material for: Role of UDP-Glycosyltransferase (ugt) Genes in Detoxification and Glycosylation of 1-Hydroxyphenazine (1-HP) in Caenorhabditis elegans
Source: Chem Res Toxicol. 2024 Mar 15;37(4):590–9. doi: 10.1021/acs.chemrestox.3c00410 (PMC11022241; doi:10.1021/acs.chemrestox.3c00410)
Supplement: Supplementary file 1 — tx3c00410_si_001.pdf [file tx3c00410_si_001.pdf]

Supplemental Information

Role of UDP-glycosyltransferase (*ugt)* genes in detoxification and glycosylation of 1-hydroxyphenazine (1-HP) in *Caenorhabditis elegans*

Muhammad Zaka Asif[1,2], Kelsey A. Nocilla[2], Li Ngo[2], Man Shah[2], Yosef Smadi[2], Zaki Hafeez[2], Michael Parnes[2], Allie Manson[2], John N. Glushka[2], Franklin E. Leach III[2,3], Arthur S. Edison[1,2,4] *

[1]Department of Biochemistry & Molecular Biology, University of Georgia, Athens, Georgia, United States 30602
[2]Complex Carbohydrate Research Center, University of Georgia, Athens, Georgia, United States 30602
[3]Department of Chemistry, University of Georgia, Athens, Georgia, United States 30602
[4]Institute of Bioinformatics, University of Georgia, Athens, Georgia, United States 30602

* Email: aedison@uga.edu

This supplement contains the following:

Item	Page
Supplemental Table 1	S2
Supplemental Table 2	S5
Supplemental Table 3	S5
Supplemental Figure 1	S7
Supplemental Figure 2	S8
Supplemental Figure 3	S9
Supplemental Figure 4	S10
Supplemental Figure 5.1	S11
Supplemental Figure 5.2	S12
Supplemental Table 5.3 (A)	S12
Supplemental Figure 5.3	S13
Supplemental Figure 6.1	S14
Supplemental Figure 6.2	S15
Supplemental Table 6.3 (A)	S15
Supplemental Figure 6.3	S16
Supplemental Figure 7	S17
Reference	S17

Supplemental Table 1. Detailed information on strains used in this study, including strain name, genotype affected, gene sequence, deletion strategy, reference article where strain is first mentioned, and time each strain takes to grow to L4.

Strain Name	Genotype	Gene Sequence	Description	Reference Article	Hours from egg to L4
N2	-	-	-	(Brenner, 1974)	~ 42 hours
PD1074	-	-	-	(Yoshimura et al., 2019)	~ 42 hours
RB2055	ugt-1(ok2718) V.	AC3.7	Homozygous. Outer Left Sequence: AGCCATGAGGACAAAGTTCG. Outer Right Sequence: TGTTGAAAATGCTTTGCCAG. Inner Left Sequence: TTGCTCTTCCATGTCTCGAA. Inner Right Sequence: TCGTCTAGATTCCGCCATTT. Inner Primer PCR Length: 1255 bp. Deletion Size: 787 bp. Deletion left flank: TTGCATTTCCAACACCATCACT TCCAAAAC. Deletion right flank: TTTGTTCAGTACTACATGTTAG ATGCTTTT.	C. *elegans* Gene Knockout Project at the Oklahoma Medical Research Foundation, International C. *elegans* Gene Knockout Consortium	~ 44 hours
VC4207	ugt-6(gk5292[loxP + myo-2p::GFP::unc-54 3' UTR + rps-	ZC455.4	Homozygous viable. Deletion of 1469 bp with Calarco/Colaiacovo selection cassette conferring myo-2 GFP and G418 resistance inserted at break. Left flanking sequence: GCGTGTTTTACCATCAGATCAG	(Au et al., 2019)	~ 42 hours

	27p::neoR::unc-54 3' UTR + loxP]) V.		TTGGCGTG ; Right flanking sequence: GATGGAATATGCTGCCTTCCAAGTTCATCA.		
VC3950	ugt-9(gk5024[loxP + myo-2p::GFP::unc-54 3' UTR + rps-27p::neoR::unc-54 3' UTR + loxP]) V.	T19H12.1	Homozygous viable. Deletion of 1266 bp with Calarco/Colaiacovo selection cassette conferring myo-2 GFP and G418 resistance inserted at break. Left flanking sequence: AAGTTTTCGGAAGGCTTTCTGTAGGGTGAA; Right flanking sequence: GGAGGTGCTGTTGCGTACGACAAATTTGAT.	-	~ 42 hours
RB2550	ugt-23(ok3541) X.	C17G1.3	Homozygous. Outer Left Sequence: cgtgacgctttagcatttca. Outer Right Sequence: tcattgatgccgatgaagaa. Inner Left Sequence: ttgatcagcgaatattggga. Inner Right Sequence: atgcacattctcatcttgcg. Inner Primer PCR Length: 1197. Estimated Deletion Size: about 300 bp.	C. *elegans* Gene Knockout Project at the Oklahoma Medical Research Foundation, International C. *elegans* Gene Knockout Consortium	~ 42 hours
PH7346	ugt-23(sb7346) X.	C17G1.3	Homozygous. Precise gene deletion (with 30bp flanks): accattcaaaaatcgaaaccattaccattc-ATGTAGTTCTCGAGTTCCCAGCACCAACATT. Strategy: CRISPR/Cas9	SUNY Biotech	~ 42 hours
RB2607	ugt-49(ok3633) V.	AC3.2	Homozygous. Outer Left Sequence: cgtgtgatggtgacaagacc. Outer Right Sequence: agaacagcaacgaacacgaa. Inner Left Sequence: acgtggcattcagtgaacaa. Inner Right Sequence: ggacaaaagcaataacatcaaga. Inner Primer PCR Length: 1279. Estimated Deletion Size: about 400 bp.	C. *elegans*	~ 44 hours

				Gene Knockout Consortium	
VC2512	ugt-60(ok3248) III/hT2 [bli-4(e937) let-?(q782) qIs48] (I;III).	C07A9.6	Homozygous lethal deletion chromosome balanced by bli-4- and GFP-marked translocation. Heterozygotes are WT with pharyngeal GFP signal, and segregate WT GFP, arrested hT2 aneuploids, and non-GFP ok3248 homozygotes (probable early larval arrest). Homozygous hT2[bli-4 let-? qIs48] inviable. Pick WT GFP and check for correct segregation of progeny to maintain. External left primer: GAAGGTTTCGGACTTGTTGC. External right primer: CGCATCCACTTTCTTCAGGT. Internal left primer: CTGAGAGCATCGCGGATAGT. Internal right primer: TGACGCGTCTAGCTCAATTTT. Internal WT amplicon: 1354 bp. Deletion size: 525 bp. Deletion left flank: TATAGCCTCCATGTGCAATCATTAATTTCA. Deletion right flank: AACCTCGATAGAACAAATTCTCGTCAACGA.	C. *elegans* Reverse Genetics Core Facility at the University of British Columbia, international C. *elegans* Gene Knockout Consortium	~ 50 hours
RB2011	ugt-62(ok2663) III.	M88.1	Homozygous. Outer Left Sequence: AAACATGGTTCCCGACATTC. Outer Right Sequence: AATTCGGTGCATTTGGAAAA. Inner Left Sequence: GCAACTTTGGAATTTTTGGG. Inner Right Sequence: ATCAGATTTCCTGCGCAACT. Inner Primer PCR Length: 3024 bp. Deletion Size: 1367 bp. Deletion left flank: ACGCTAAATTGTTTTAATACATTTTAAAGT. Deletion right flank: ATGAAATATTTCTCGATTAAAGTTTCTCAG.	C. *elegans* Gene Knockout Project at the Oklahoma Medical Research Foundation, International C. *elegans* Gene Knockout Consortium	~ 46 hours

VC4339	ugt-66(gk5422[loxP + myo-2p::GFP::unc-54 3' UTR + rps-27p::neoR::unc-54 3' UTR + loxP]) III.	C23G10.6	Deletion of 2473 bp with Calarco/Colaiacovo selection cassette conferring myo-2::GFP and G418 resistance inserted at break. Left flanking sequence: AAAATTTCAAAATATTAAATGAAGCCGTTG; Right flanking sequence: CAGGGAGGTGTCACAATTATTTGTGTCCTG.	(Au et al., 2019)	~ 42 hours
RB1342	ogt-1(ok1474) III.	K04G7.3	Homozygous. Outer Left Sequence: gccaaagaattgatttcgga. Outer Right Sequence: tgctcttgcaccacaaccta. Inner Left Sequence: acctgtccgagaccattctg. Inner Right Sequence: ccaacgctattgctcctctc. Inner Primer PCR Length: 2730. Estimated Deletion Size: about 1300 bp.	C. *elegans* Gene Knockout Project at the Oklahoma Medical Research Foundation, International C. *elegans* Gene Knockout Consortium	~ 43 hours

Note: All strains were obtained from Caenorhabditis Genetics Center (CGC).

Supplemental Table 2. Assigned molecular compositions in high-resolution MS data for 1-HP derived C. elegans metabolites acquired using positive-ion electrospray ionization (ESI).

**Compound**	**Ion**	**Ion Formula**	**Calculated *m/z***	**Observed *m/z***
b-D-glucopyranosyl-phenazine	$[M+H]^+$	$C_{18}H_{19}N_2O_6^+$	359.1237	359.1240
b-D-glucopyranosyl (1-6)-b-D-glucopyranosyl-phenazine	$[M+H]^+$	$C_{24}H_{29}N_2O_{11}^+$	521.1766	521.177
b-D-glucopyranosyl (1-6)- [b-D-glucopyranosyl (1-2)]-b-D-glucopyranosyl-phenazine	$[M+H]^+$	$C_{30}H_{39}N_2O_{16}^+$	683.2294	683.2305
b-D-glucopyranosyl (1-6)- [b-D-N-acetylglucosamine-pyranose (1-2)]-b-D-glucopyranosyl-phenazine.	$[M+H]^+$	$C_{32}H_{42}N_3O_{16}^+$	724.2560	724.2559

Note: Representative data is shown for N2.

Supplemental Table 3. Assigned fragment ions in high-resolution MS/MS data for 1-HP-derived *C. elegans* metabolites acquired using positive-ion electrospray ionization (ESI).

Compound 5

Ion	Theoretical m/z	Observed m/z	Mass Measurement Error (ppm)
HexNAc-2Hex-1HP	724.2559	724.255	1.24
HexNAc-1Hex-1HP	562.2031	562.2037	-1.07
HexNAc-2Hex	528.1923	528.1925	-0.38
2Hex-1HP	521.1765	521.177	-0.96
HexNAc-Hex	366.1395	366.1401	-1.64
Hex-1HP	359.1237	359.1241	-1.11
HexNAc	204.0867	204.0871	-1.96
1HP	197.0709	197.0713	-2.03
1Hex	163.0601	163.0602	-0.61

Compound 4

Ion	Theoretical m/z	Observed m/z	Mass Measurement Error (ppm)
3Hex-1HP	683.2293	683.2305	-1.76
2Hex-1HP	521.1765	521.177	-0.96
1Hex-1HP	359.1237	359.1244	-1.95
2Hex	325.1129	325.1136	-2.15
1HP	197.0709	197.0713	-2.03
1Hex	163.0601	163.0603	-1.23

Compound 3

Ion	Theoretical m/z	Observed m/z	Mass Measurement Error (ppm)
2Hex-1HP	521.1765	521.1768	-0.58
Hex-1HP	359.1237	359.1245	-2.23
1HP	197.0709	197.0713	-2.03
1Hex	163.0601	163.0603	-1.23

Compound 2

Ion	Theoretical m/z	Observed m/z	Mass Measurement Error (ppm)
Hex-1HP	359.1237	359.1238	-0.28
1HP	197.0709	197.0716	-3.55

Note: Representative data is shown for N2.

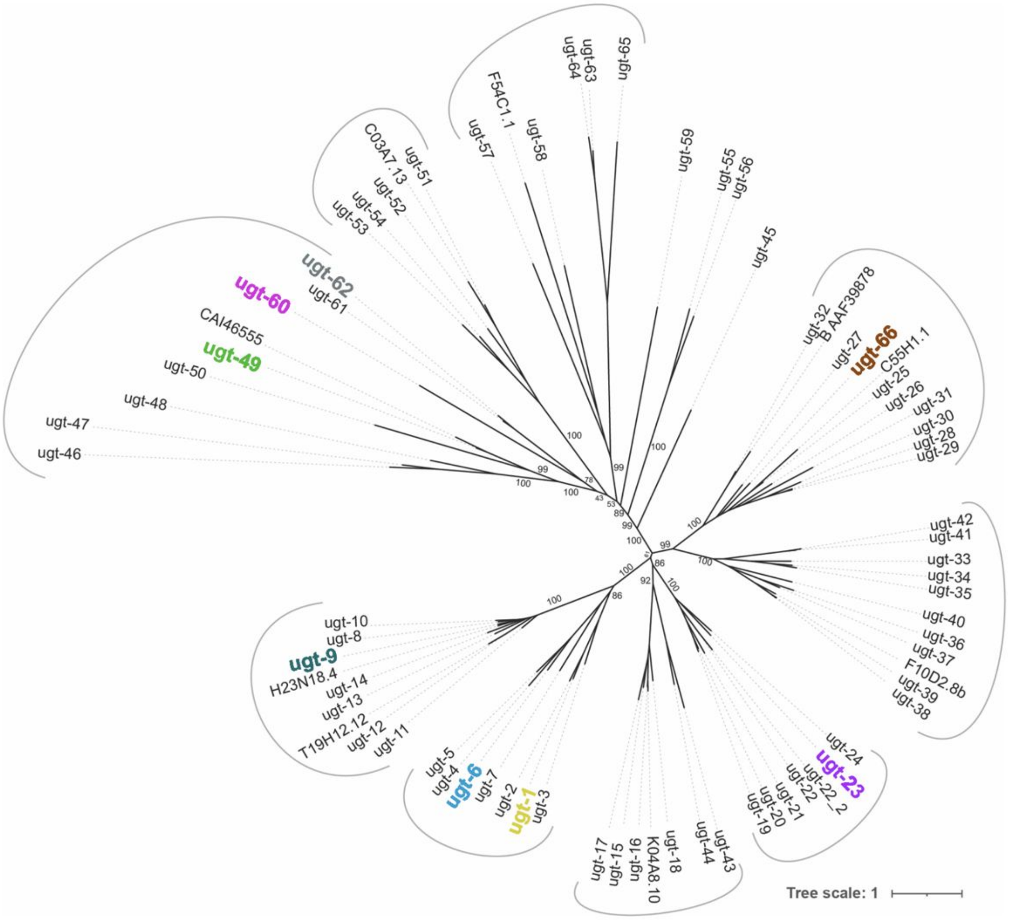

Supplemental Figure 1. Phylogenetic tree highlighting *ugt* genes used in this study.[1] As we showed previously, *C. elegans* has over 70 *ugt*s, and they form about 9 distinct clades. The specific *ugt*s used for this study cover 5 of these clades. The information in this phylogeny can be used to help prioritize future work on more *ugt* genes to better understand their role in detoxification pathways. This was published under terms of the Creative Commons Attribution 4.0 International license.

A

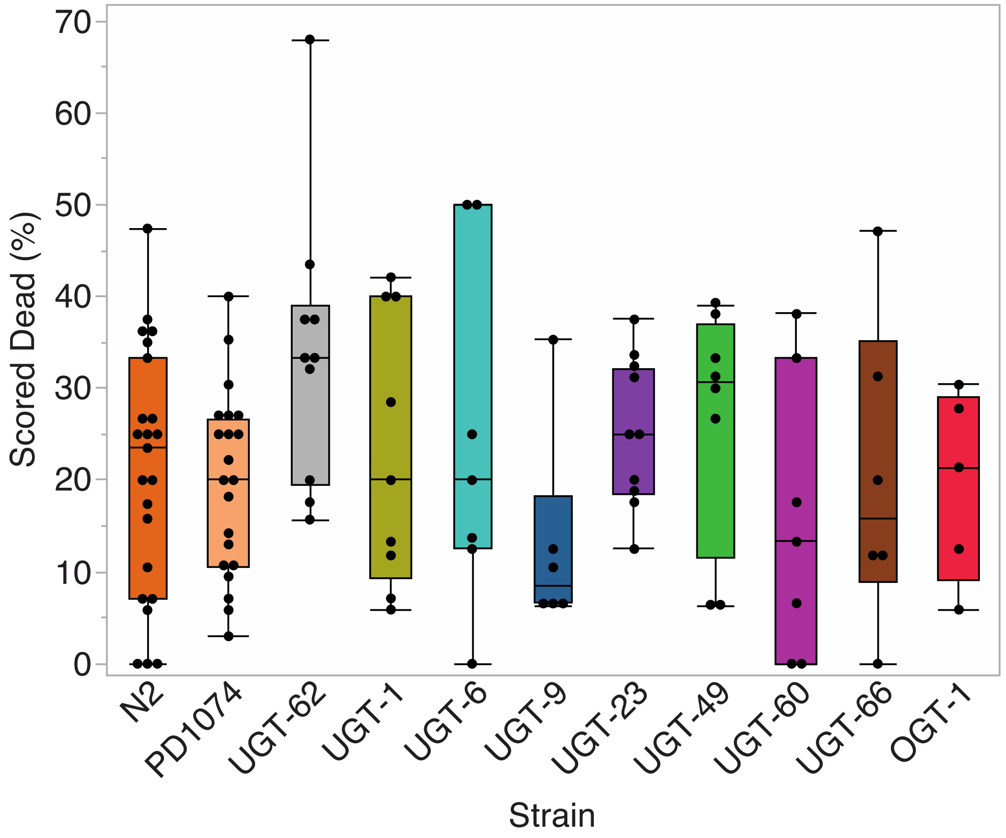
Scored Dead (%)
70
60
50
40
30
20
10
0
N2
PD1074
UGT-62
UGT-1
UGT-6
UGT-9
UGT-23
UGT-49
UGT-60
UGT-66
OGT-1
Strain

B

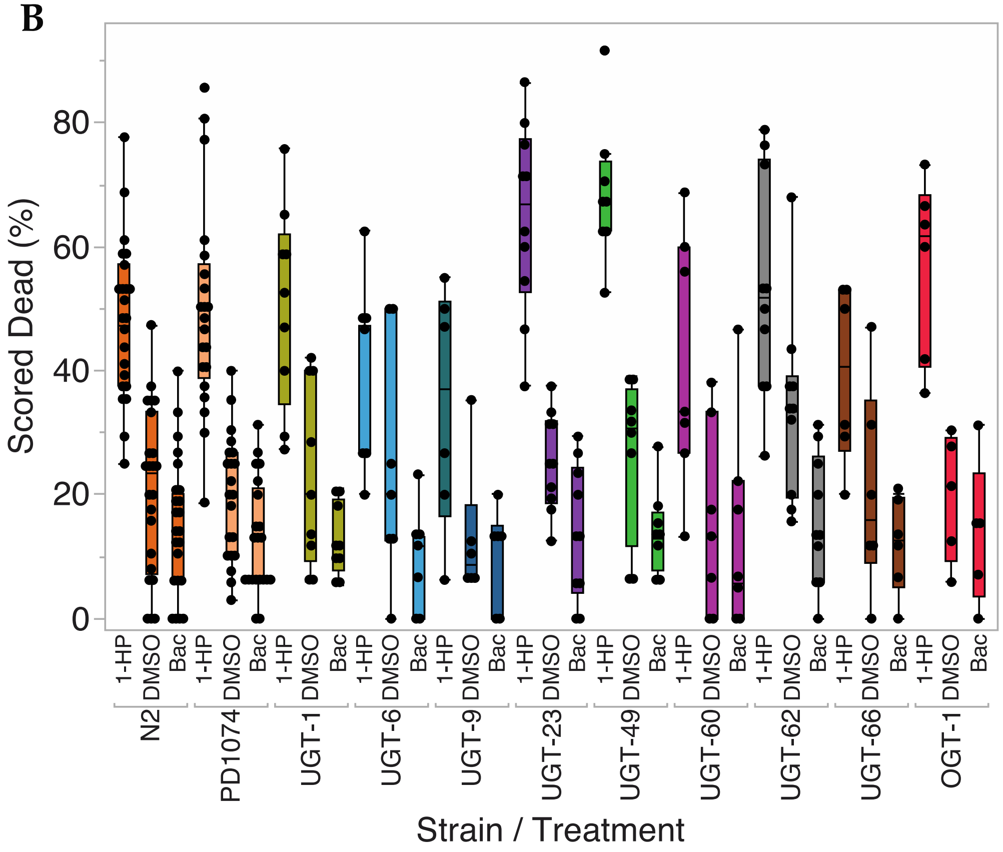
Scored Dead (%)
80
60
40
20
0
1-HP DMSO Bac
N2
PD1074
UGT-1
UGT-6
UGT-9
UGT-23
UGT-49
UGT-60
UGT-62
UGT-66
OGT-1
Strain / Treatment

C

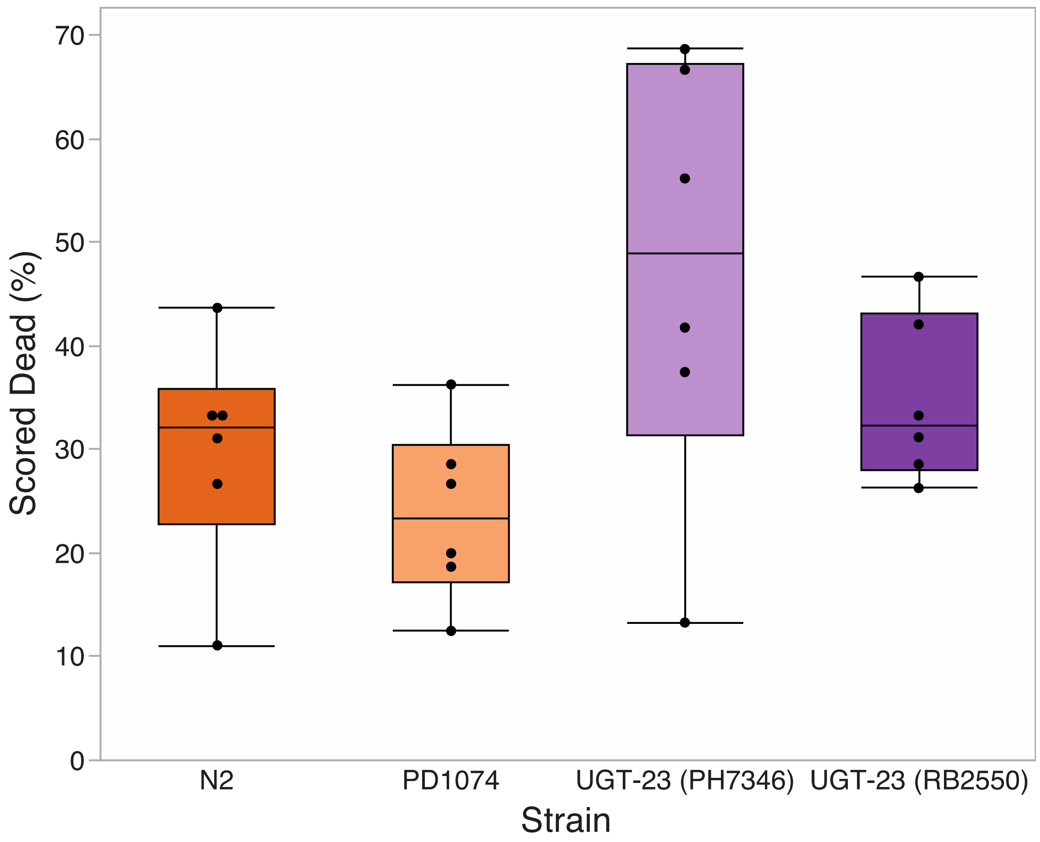

Supplemental Figure 2. Percentage of worms scored in plate-based mortality screen after 7 h incubation. (A) Box and whiskers plot showing mortality of various strains to 1.1% DMSO for 7h (n >/= 6). (B) Box and whiskers plot showing combined data of exposure to 179µM 1-HP, 1.1% DMSO and bacteria control showing increased mortality upon 1-HP exposure for each strain compared to controls after Tukey's HSD test ($\alpha = 0.05$). (C) Box and whiskers plot comparing mortality of the CGC *ugt-23* knockout strain (RB2550) and the strain generated by SUNY biotech (n=6). The p-value between the two strains is 0.22 indicating no significant difference in mortality.

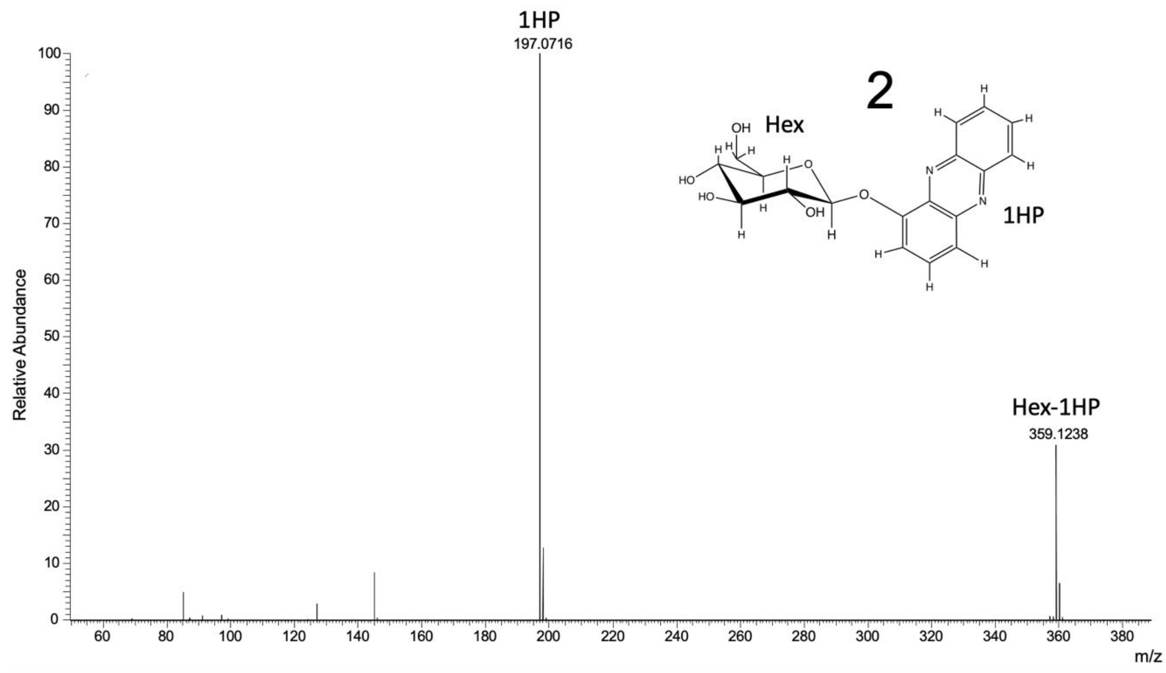

Supplemental Figure 3. MS/MS data for (2).

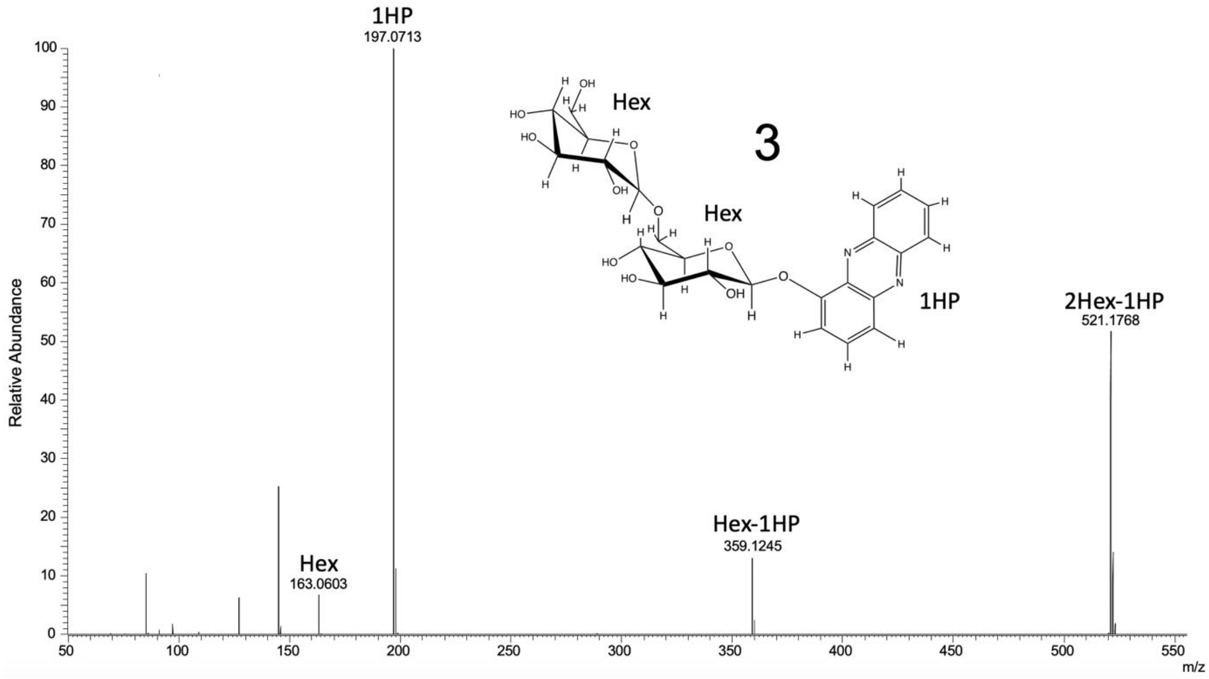

Supplemental Figure 4. MS/MS data for (3).

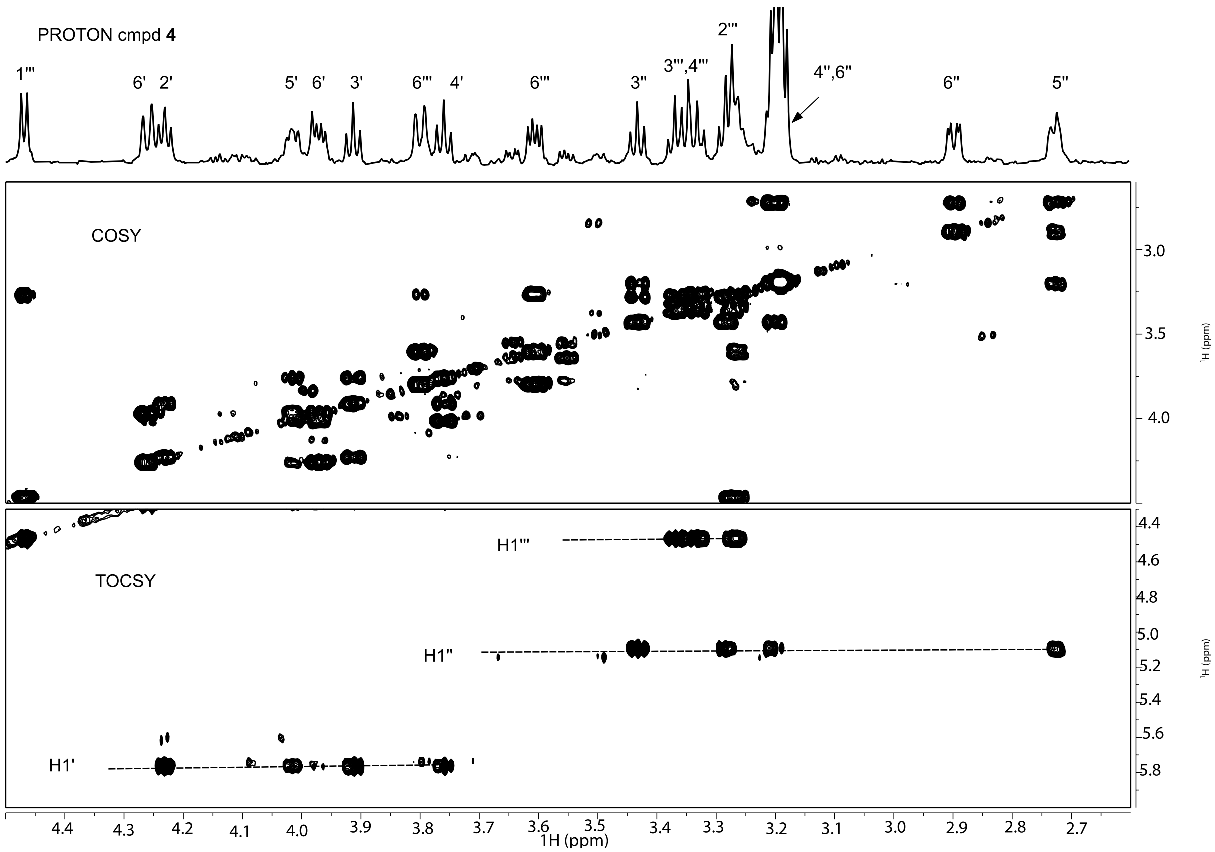

Supplementary Figure 5.1: Proton NMR assignments of compound (**4**) glucosyl residues.

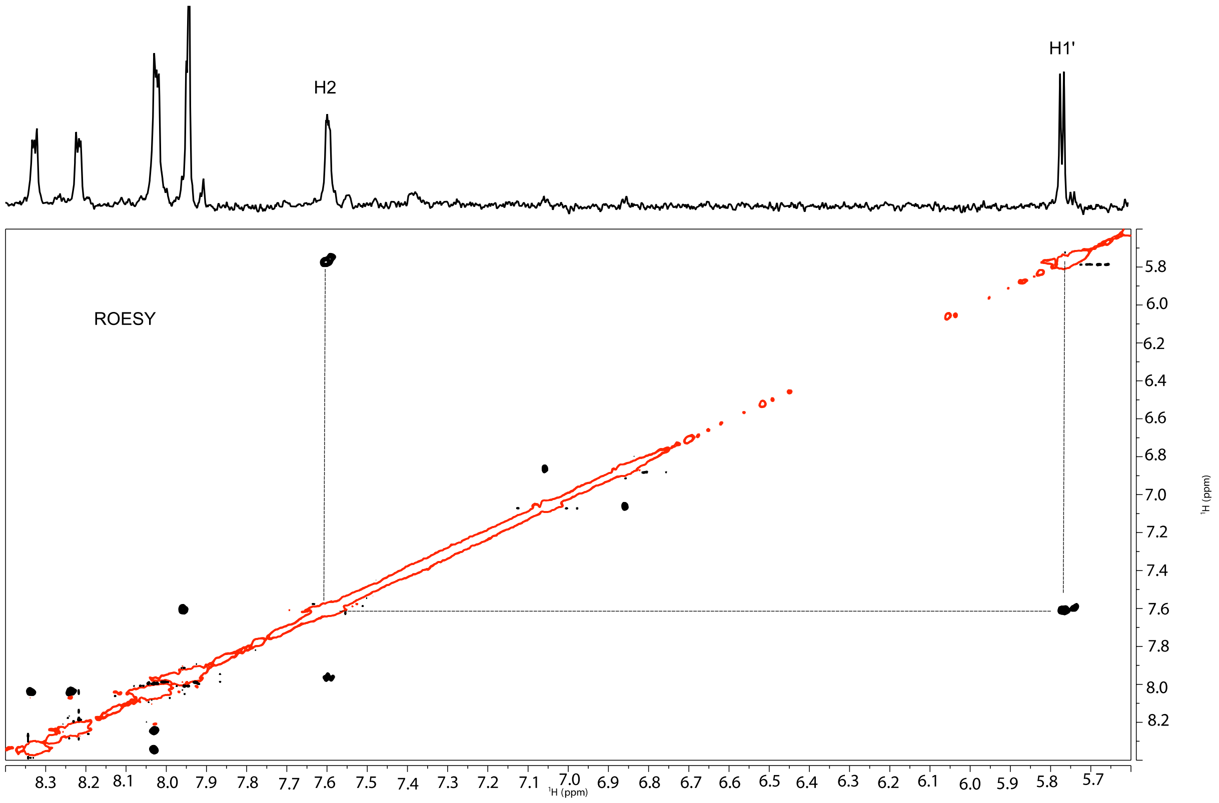

Supplementary Figure 5.2: Top panel – the region of 1D proton of compound (**4**) showing phenazine protons and H1'. Bottom panel – the region of 2D ROESY spectrum showing NOE between H2 of phenazine and H1' of the glucosyl residue linked to position 1 of phenazine.

Supplemental Table 5.3 (A): NMR data table showing assignments of Compound (4).

A

#	Type	d_H [ppm]	J (Hz)
2	dd	7.59	6.02, 2.94
3-4	dd	7.94	
6	ddd	8.33	
7-8	ddd	8.02	
9	ddd	8.21	
1'	d	5.76	7.59
2'	t	4.23	8.16
3'	t	3.91	8.26
4'	t	3.76	10.46
5'	t	4.01	8.44
6a'	m	3.97	
6b'	d	4.26	11.21
1''	d	5.1	7.68
3''	t	3.43	9.3

4’’	m	3.19	
5’’	t	2.73	
6a’’	dd	2.89	12.3, 4.22
6b’’	m	3.19	
1’’’	d	4.47	7.71
2’’’	m	3.27	
3’’’	m	3.35	
4’’’	m	3.35	
6a’’’	dd	3.61	6.21, 12.41
6b’’’	d	3.8	11.95

B

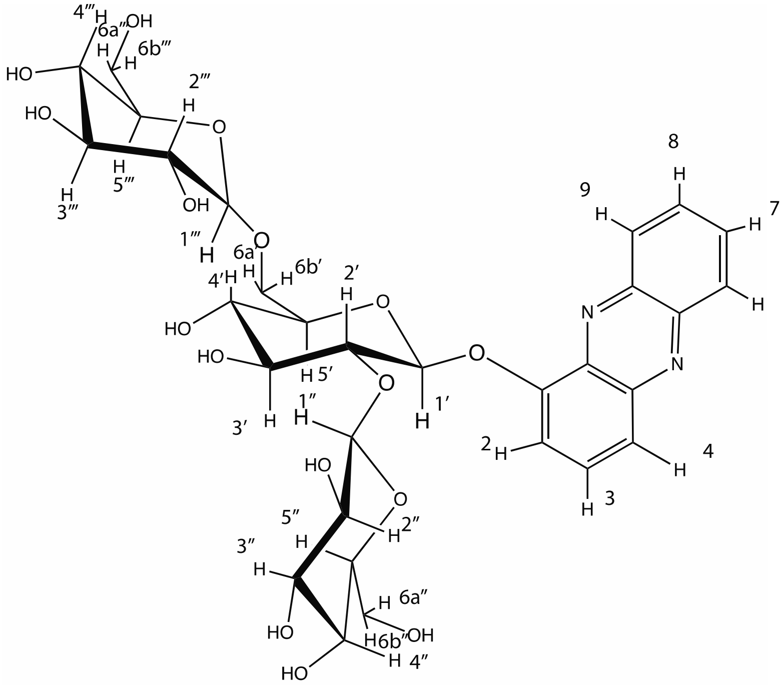

Supplemental Figure 5.3: NMR data of compound (**4**) in D_2O. (A) Table showing assignments. d indicates a doublet, dd indicates a doublet of doublets, ddd indicates doublet of doublet of doublets, t indicates a triplet, and m indicates a multiplet. (B) Structure of compound (**4**).

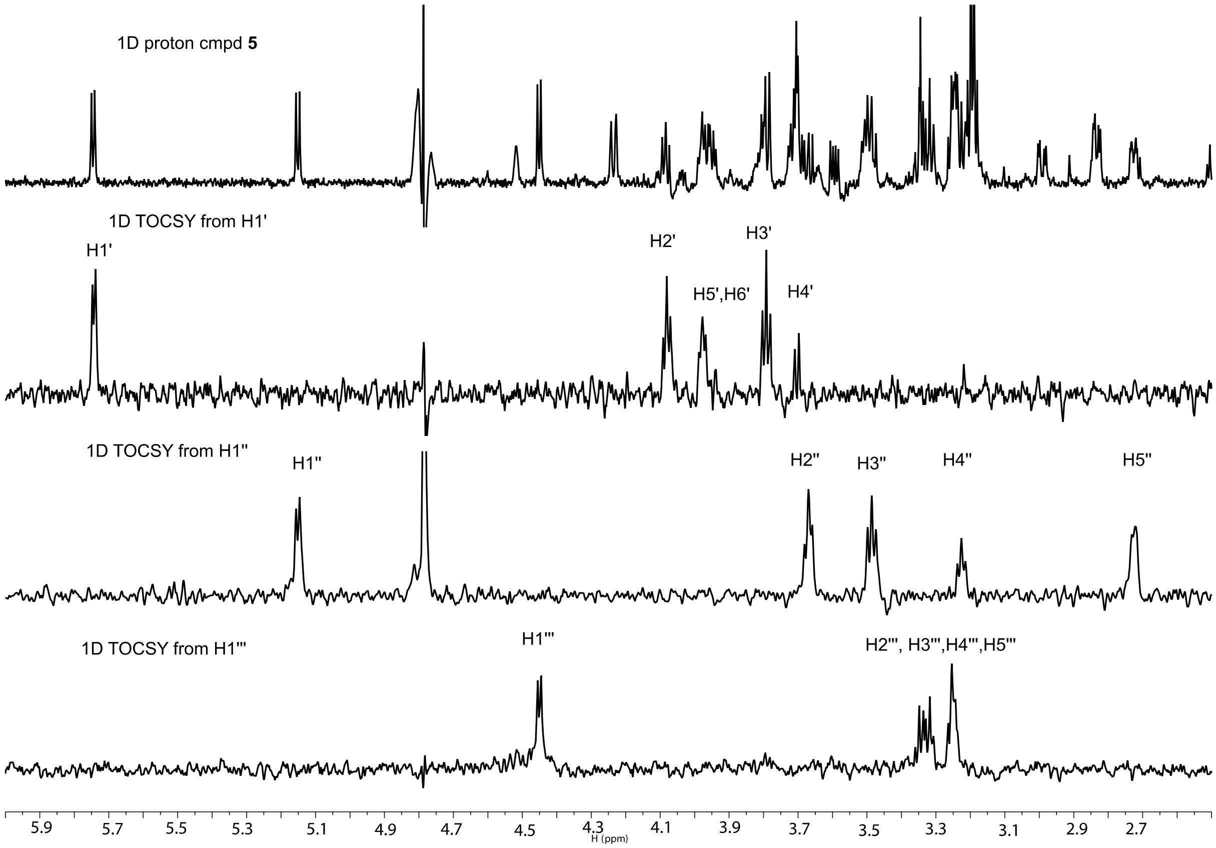

Supplementary Figure 6.1: Proton NMR spectra of compound (**5**). The three lower panels of 1D TOCSY spectra show signals from H1-H5 for the two glucosyl residues and the NAcetyl-glucosamine residue.

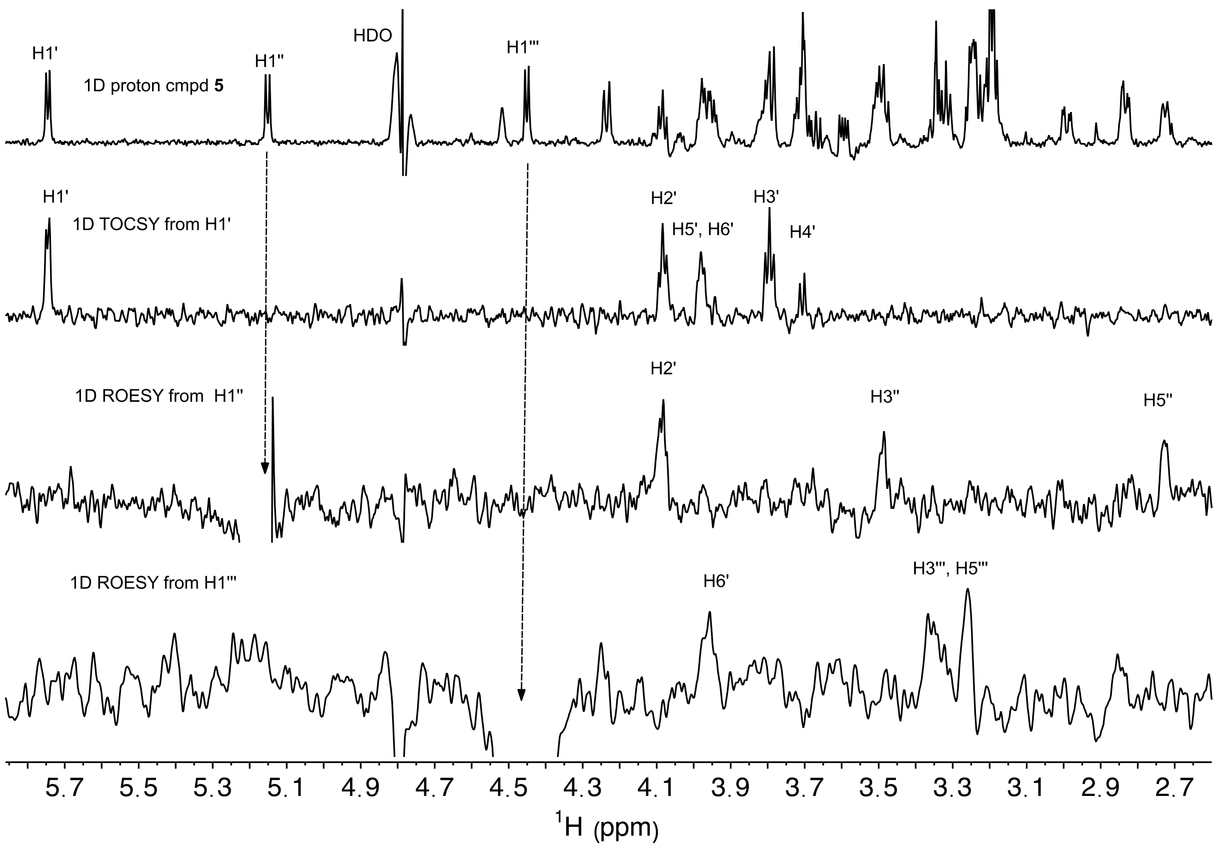

Supplementary Figure 6.2: Regions of 1D TOCSY and ROESY spectra of compound (**5**) confirm the terminal glucose and NAcetyl-glucosamine linkages to positions 2' and 6' of the glucosyl residue attached to phenazine. The arrows indicate the peaks of interest that have been selected for the 1D ROESY experiment. They correspond with the anomeric protons of each of the sugar residues of the compound.

Supplemental Table 6.3 (A): NMR data table showing assignments of Compound (5).

A

#	Type	δ_H [ppm]	J (Hz)
2	dd	7.59	6.29, 1.91
3-4	dd	7.96	
6	ddd	8.34	
7-8	ddd	8.04	
9	ddd	8.26	
1'	d	5.75	7.41
2'	t	4.08	8.16
3'	m	3.79	
4'	m	3.68	
5'	m	3.95	

6’	m	3.96	
1’’	d	5.15	8.32
2’’	dd	3.66	
3’’	m	3.49	
4’’	m	3.24	
5’’	ddd	2.72	
1’’’	d	4.45	7.7
2’’’	m	3.33	
3’’’	m	3.33	
4’’’	m	3.33	
5’’’	m	3.24	

B

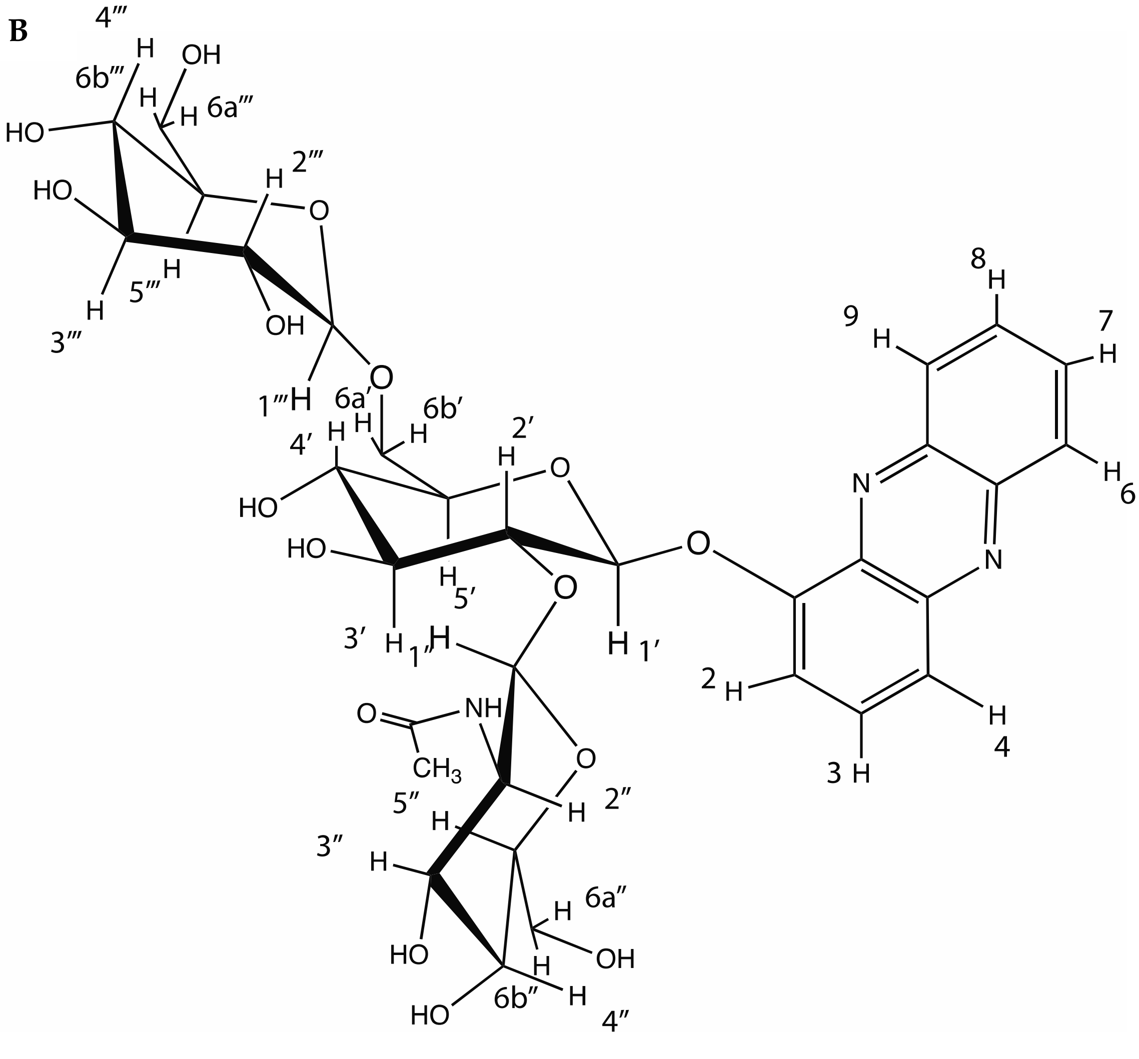

Supplemental Figure 6.3: NMR data of compound (**5**) in D_2O. (A) Table showing assignments. d indicates a doublet, dd indicates a doublet of doublets, ddd indicates doublet of doublet of doublets, t indicates a triplet, and m indicates a multiplet. (B) Structure of compound (**5**).

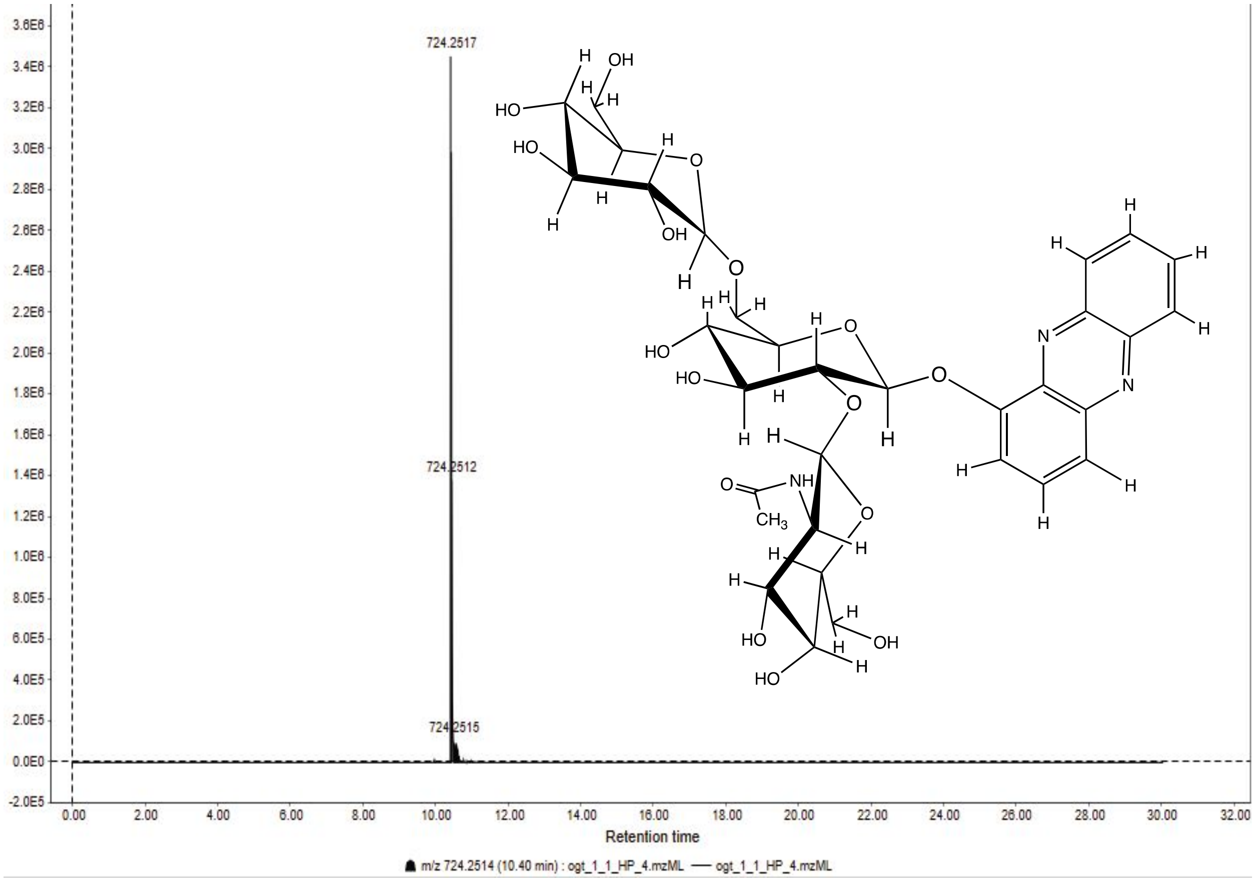

Supplemental Figure 7. Selected ion chromatogram for Compound (**5**) in the *ogt-1* knockout mutant exposed to 22.3 μM 1-HP.

Reference:

(1) Asif, M. Z.; Benveniste, M. C.; Chism, K. D.; Levin, A. L.; Lanier, D.; Watkins, R. E.; Taujale, R.; Tucker, N.; Edison, A. S. Computational analysis of variation in C. elegans ugts. *MicroPubl Biol* **2023**, *2023*. DOI: 10.17912/micropub.biology.000819 From NLM PubMed-not-MEDLINE.
